# Proteomic Profiling Unravels a Key Role of Specific Macrophage Subtypes in Sporadic Inclusion Body Myositis

**DOI:** 10.3389/fimmu.2019.01040

**Published:** 2019-05-09

**Authors:** Andreas Roos, Corinna Preusse, Denisa Hathazi, Hans-Hilmar Goebel, Werner Stenzel

**Affiliations:** ^1^Department of Neuropediatrics, Developmental Neurology and Social Pediatrics, Centre for Neuromuscular Disorders in Children, University Children's Hospital Essen, University of Duisburg-Essen, Essen, Germany; ^2^Leibniz-Institut für Analytische Wissenschaften -ISAS- e.V., Dortmund, Germany; ^3^Department of Neuropathology, Charité -Universitätsmedizin Berlin, Corporate Member of Freie Universität Berlin, Humboldt-Universität zu Berlin, and Berlin Institute of Health, Berlin, Germany; ^4^Leibniz Science Campus Chronic Inflammation, Berlin, Germany

**Keywords:** CD74, SIGLEC1, CD163, STAT1, STAT6, type I interferon (IFN), muscle proteomics

## Abstract

Unbiased proteomic profiling was performed toward the identification of biological parameters relevant in sIBM, thus giving hints about the pathophysiological processes and the existence of new reliable markers. For that purpose, skeletal muscle biopsies from 13 sIBM and 7 non-diseased control patients were analyzed with various methods, including liquid chromatography coupled to tandem mass spectrometry (four patients). Subsequent data analysis identified key molecules further studied in a larger cohort by *q*PCR, immunostaining, and immunofluorescence *in situ*. Proteomic signature of muscle biopsies derived from sIBM patients revealed the chaperone and cell surface marker CD74, the macrophage scavenger molecule CD163 and the transcription activator STAT1 to be among the highly and relevantly expressed proteins suggesting a significant contribution of immune cells among the myofibers expressing these markers. Moreover, *in silico* studies showed that 39% of upregulated proteins were involved in type I or mixed type I and type II interferon immunity. Indeed, further studies via immunohistochemistry clearly confirmed the prominent involvement of the key type I interferon signature-related molecules, ISG15 as well as IRF8 with MHC class II^+^ myofibers. Siglec1 colocalized with CD163^+^ macrophages and MHC class II molecules also co-localized with CD74 on macrophages. STAT1 co-localized with Siglec1^+^ macrophages in active myofibre myophagocytosis while STAT6 colocalized with endomysial macrophages. These combined results show involvement of CD74, CD163, and STAT1 as key molecules of macrophage activation being crucially involved in mixed and specific type I interferon, and interferon gamma associated-pathways in sIBM. On a more general note, these results also highlight the type of immune-interaction between macrophages and myofibers in the etiopathology of sIBM.

## Introduction

Inclusion body myositis is a chronic muscle-specific disease of adulthood leading to progressive and very characteristic hip flexor and quadriceps paresis, long finger flexor paresis, and swallowing difficulties (1–4). Muscle biopsy reveals a severe myopathic/dystrophic process with the characteristic complex inflammatory infiltrate composed of different types of T cells, macrophages and other mononuclear cells. Additional degenerative changes with the presence of amyloidogenic protein deposits, disturbed autophagy, and mitochondrial abnormalities are present. Clinical and muscle biopsy characteristics are the basis of a precise diagnosis of sIBM (1, 3, 5, 6). The autoantibody cN1A can be used as a marker of severity in sIBM, however, its role in the pathogenicity of this disease has not been fully elucidated (7–9). Despite the characteristic clinical picture and well-known biopsy findings, the pathogenesis of sIBM is still elusive and not fully understood. So far, therapeutic approaches have not been broadly successful, questioning the (auto)-immune pathogenesis. Moreover, these therapeutic interventions present attempts to address the degenerative autophagic dysfunction (10).

Proteomics and subsequent specific data analysis of diseased tissue such as skeletal muscle can help to identify key pathogenic molecules or groups of molecules involved in certain processes, which may be of relevance in inflammatory or genetic diseases affecting muscle fiber integrity [exemplified in (11)].

In this study, we applied unbiased proteomic profiling and identified CD74, CD163, and STAT1 among the highly expressed proteins in muscle biopsies of sIBM patients. Notably, these proteins were found to localize to macrophages and partially to the sarcolemma of myofibers. Further analyses were performed in the larger context of associated immune responses in skeletal muscle tissue. These approaches revealed a specific and key role of the cellular interaction of specifically activated macrophages with myofibers.

## Materials and Methods

### Patients

Clinical data of all IBM patients enrolled in this study are listed in Table 1. We included patients with clinical, and morphological signs and symptoms consistent with sIBM, according to present criteria (12), as well as sex- and age-matched patients defined as non-diseased controls (NDCs). We chose as controls subjects who had undergone a muscle biopsy, but who were found not to have any inflammatory muscle disease. They had suffered from non-specific complaints like myalgia, but objective muscle weakness and morphological abnormalities on skeletal muscle biopsy were absent. CK levels were normal and no signs of systemic inflammation and no myositis-specific antibodies (MSA) or myositis-associated antibodies (MAA) were detectable. sIBM patients had moderate illness (still ambulatory) and homogeneous muscle biopsy findings (not severely atrophic muscle bulk). Informed consent was obtained from all patients and the Charité ethics committee (EA2/163/17), had granted ethical approval.

**Table 1:** Summarized clinical information of all IBM patients included in the study.

**Patient No**.	**Age**	**Sex**	**Duration of disease (years)**	**Muscle symptoms**
sIBM1	72	M	?	Symmetric LL prox. and distal paresis, CK 3-fold elevated
sIBM2	78	F	3	Prox. LL and paresis of finger flexors, CK 2-fold elevated
sIBM3	64	M	2	Prox. tetraparesis and paresis of distal forearms, CK 2-fold elevated
sIBM4	68	F	3	Chronic progressive tetraparesis, significant muscle atrophy
sIBM5	66	M	5	Prox. paresis LL, significant atrophy of vastus lateralis
sIBM6	75	F	?	Prox. paresis LL and distal paresis UL, CK 6-fold elevated
sIBM7	64	M	?	Prox. paresis and distal paresis of fingers
sIBM8	75	M	2	Prox. progressive paresis LL and exercise induced pain, CK 3-fold elevated
sIBM9	66	M	?	Dysphagia, distal and proximal tetraparesis, CK 1.5-fold elevated
sIBM10	79	F	11	Prox. tetraparesis, progressive muscle atrophy
sIBM11	76	M	>2	Muscle pain, proximal weakness, muscle atrophy, CK normal
sIBM12	72	F	7	Prox. tetraparesis, muscle atrophy
sIBM13	71	M	?	Prox. tetraparesis

### Skeletal Muscle Specimens

In this study, we analyzed skeletal muscle biopsies derived from sIBM patients (clinically and morphologically definite sIBM) (12). Skeletal muscle biopsies from sIBM patients were used to produce proteomic results (four biopsies) and qualitative morphological characteristics *in situ* (whole cohort). In addition, four control muscle biopsies were included for the proteomic profiling and additional nine biopsies for subsequent immunohistochemical and *q*PCR studies. All skeletal muscle specimens were cryopreserved at −80°C prior to analysis.

### Morphological Analysis

All stains were performed on 7 μm cryomicrotome sections, according to standard procedures. Immunohistochemical and double immunofluorescence reactions were carried out as described previously (13). The following antibodies were used for staining procedures:

Mouse anti-human CD163, 1:50, St. John's Lab/polyclonal; rabbit anti-human CD74, 1:100 St. John's Lab/polyclonal; mouse anti-human CD68, 1:100 Dako/EBM1; rabbit anti-human iNOS, ready-to-use, Genetex/polyclonal; rabbit anti-human ISG15, 1:100, abcam/polyclonal; MHCI, 1:1.000, Dako/W6/32; mouse anti-human MHC class II, 1:100, DAKO/ CR3/43; rat anti-human STAT1, 1:50, R&D Systems/246523; mouse anti-human STAT6, 1:50, R&D Systems/253906; mouse anti-human Siglec1, 1:100, Novus Biologicals/HSn7D2; IRF8, 1:100 Abcam/polyclonal.

### Proteomics

Proteomic profiling of four sIBM-patient derived and four control quadriceps muscles was carried out as described previously (14).

### *In Silico* Studies

Further *in silico* studies included “Proteomaps” (www.proteomaps.net), “Interferome” (www.interferome.org), “Cytoscape” (www.cytoscape.org) and “STRING” (www.string-db.org) and have been carried out to unravel functional connections and interdependences between the proteins vulnerable in sIBM with a special focus on such involved in the interferon-mediated immune response. All regulated proteins (24 down, 119 up-regulated) were used for the analyses.

“Proteomaps” enables us to obtain a picture of the quantitative composition of vulnerable pathways and cellular processes with a focus on individual protein functions controlling these pathways and processes. The visualization of affected pathways (and responsible proteins) is built automatically from the computerized proteome data and based on the “KEGG Pathways” gene classification. Hereby, individual proteins are shown as polygons and to emphasize the fold of regulation, polygon-sizes reflect fold of changes abundances. Functionally related proteins are arranged in proximity. This *in silico* tool has been applied to proteins showing a statistically significant altered abundance and thus vulnerability in sIBM-diseased muscle. “Interferome” enables the reliable identification of individual interferon-regulated genes or respective molecular signatures. Here, “Ensembl IDs” have been utilized to filter for interferon-regulated genes (or rather corresponding proteins) based on our proteomic findings. “Cytoscape” as an additional open source *in silico* tool enabling the visualization of molecular and functional protein-protein interaction networks. Here, we applied “Cytoscape” to proteins modulated by the interferon-response (based on the results of our “Interferome”-based data analysis). “STRING“ (Search Tool for the Retrieval of Interacting Genes/Proteins) represents an *in silico* tool enabling the delineation of (direct and functionally related) protein-protein interactions and thus allows to identify functional interdependences of proteins with altered abundances in diseased tissues such as sIBM muscle. Here, we applied “STRING” to decipher proteins interacting with CD74, STAT1, and CD63.

### Quantitative Reverse Transcription PCR (*q*RT-PCR)

Total RNA was extracted from muscle specimens using the technique described previously (13). Briefly, cDNA was synthesized using the High-Capacity cDNA Archive Kit (Applied Biosystems, Foster City, CA). For qPCR reactions, 2 ng of cDNA were used and for subsequent analysis, the 7900HT Fast Real-Time PCR System (Applied Biosystems, Foster City, CA) was utilized with the following, running conditions: 95°C 0:20, 95°C 0:01, 60°C 0:20, 45 cycles (values above 40 cycles were defined as not expressed). All targeted transcripts were run as triplicates. For each of these runs, the reference gene *PGK1* has been included as internal control to normalize the relative expression of the targeted transcripts. The qPCR assay identification numbers, TaqMan® Gene Exp Assay from Life Technologies/ThermoFisher are listed as follows: STAT1 Hs01013989_m1, STAT6 Hs00598625_m1, PGK1 Hs99999906_m1. The ΔCT of non-diseased controls was subtracted from the ΔCT of sIBM patients muscles to determine the differences (ΔΔCT) and fold change (2^∧^−ΔΔCT) of gene expression. Gene expression was illustrated by the log10 of fold change values compared to NDCs.

### Statistical Analysis

Statistical analysis of proteomic data has been carried out as described previously (14). Kruskal-Wallis one-way ANOVA followed by Bonferroni-Dunn correction of the *post hoc* tests was used to analyze quantitative differences of mRNA transcripts. Data are presented as mean ± SEM. The level of significance was set at *P* < 0.05. GraphPad Prism 5.02 software (GraphPad Software, Inc., La Jolla, CA, USA) was used for statistical analysis.

## Results

### Proteomic Signature: CD74, CD163, and STAT1 Are Highly Expressed in Skeletal Muscle Biopsies of sIBM Patients

**Figure 1:**
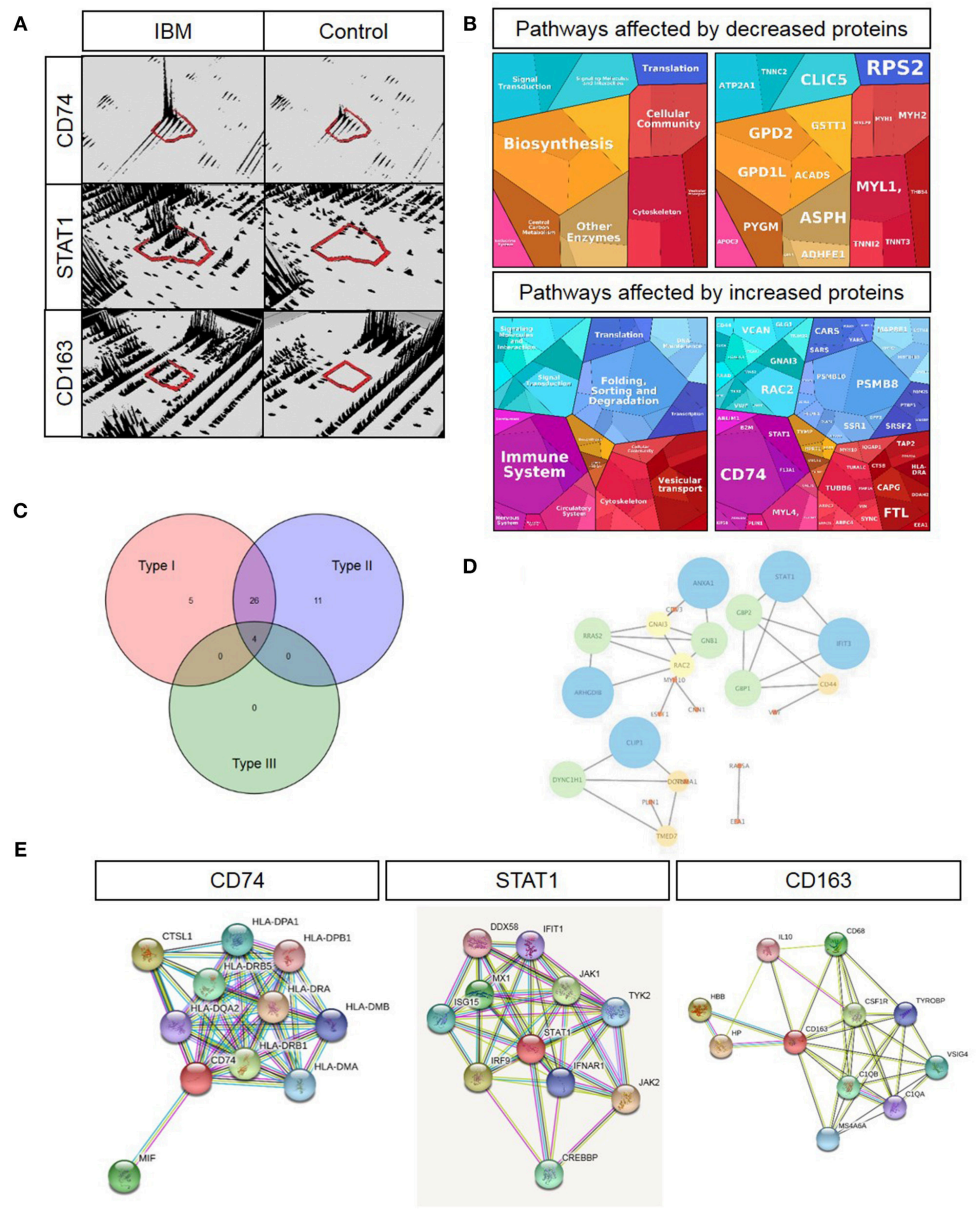
Key proteomic findings and subsequent *in silico* analyses. **(A)** 3-D montages of representative tryptic peptides highlighting the increased abundance of the corresponding proteins, CD47, STAT1, and CD163. **(B)** Proteomaps-based analysis of cellular processes addressed by up- and down-regulated proteins in sIBM-patient-derived muscle (left figures) as well as of proteins predominating the respective cellular processes by taking their relative abundance/fold of regulation into consideration (right figures). **(C)** Venn diagram-based categorization of proteins increased in sIBM-patient-derived muscle and modulated by the three different interferon types (detailed list of increased proteins controlled by interferons is provided in Table 2). **(D)** Cytoscape-based visualization of functional networks of upregulated proteins controlled by interferons. **(E)** STRING-based analysis of functional protein networks of CD74 (left figure), STAT1 (figure in the middle) and CD163 (right figure) toward the identification of further interferon-modulated and macrophage-expressed proteins with pathophysiological significance in sIBM.

Since the precise pathogenesis of IBM is still unclear, we aimed to analyze the proteomic signature via label-free profiling as an unbiased approach to gather new relevant molecules that might play decisive roles in the disease pathogenesis. The proteomic analysis unraveled that CD74 (6.7-fold; log_2_-ratio), STAT1 (5-fold; log_2_-ratio), and CD163 (4.8-fold; log_2_-ratio) are among the highly expressed proteins in skeletal muscle specimens derived from sIBM patients (Figure 1A). *In silico* studies (proteomap: https://www.proteomaps.net/) of all proteins altered in abundance (out of 1375 quantified proteins, 24 are statistically significantly decreased and 119 are increased; vulnerability of 10.4% of the investigated proteome) in sIBM-patient derived skeletal muscles, revealed alteration of biosynthesis, cellular composition, cytoskeleton and altered protein processing (folding, sorting, and degradation) along with vesicular transport. Moreover, the activation of the immune response is a predominating mechanism mirrored by an increase in specific proteins (Figure 1B). Remarkably, proteomap-based linking of altered cellular processes to key proteins (taking their fold of regulation into consideration) revealed that CD74 and STAT1 hereby seem to be major molecular “determinators” of modulation of the immune response. Prompted by this finding and by the fact that CD74, STAT1, and CD163 are well-known modulators of interferon-mediated processes (15–21), all up-regulated proteins (total of 119 proteins) were additionally analyzed *in silico* using the “interferome” platform (http://www.interferome.org). This resulted in the identification of a total 46 transcripts corresponding to the proteins up-regulated in the muscle of sIBM patients which are controlled by interferon-modulated processes (Figure 1C). Hereby 26 out of the 46 up-regulated transcripts are controlled by both types I and II interferon and four proteins by types I, II, and III. Five transcripts are controlled either by type 1 interferons while eleven are by type 2 interferons (Figure 1C). An additional analysis of the interferon-pathway controlled proteins via the “cytoscape” platform (https://cytoscape.org/) confirmed a functional interdependence of these proteins (Figure 1D), thus suggesting a functional relevance in sIBM-pathogenesis.

A further STRING analysis (https://string-db.org) toward the identification of functional protein-protein networks confirmed the functional interplay of the proteins encoded by these transcripts (Figure 1D), suggesting a fundamental role of interferon-modulated processes in the etiopathology of sIBM. An individual STRING analysis of CD74, STAT1, and CD163 confirmed a functional link of these immune-response proteins to a variety of interferon-induced proteins (Figure 1E).

Based on the dense networks of predicted functional protein-protein interplays, we evaluated the potential reaction partners and hence decided to focus on some key players for further studies. The selected key players are MIF—Macrophage migration inhibitory factor, involved in the interferon type response and showing a functional interplay with CD74 (Figure 1E) as well as ISG15, which was not upregulated in our analyses, but represents a well-known partner (22) for e.g., STATs, JAKs and IL-6, which are all found in our STRING analysis (Figure 1E). Both of these molecules showed an increased immunoreactivity within macrophages in sIBM-patient derived muscle biopsy specimens, thus supporting the concept of the involvement of different types of interferon-mediated downstream intracellular activation programs in macrophages. The concept of a particular role of macrophage-mediated interferon response in sIBM is not only further supported by increased abundance of Macrophage-capping protein (CAPG; identified via proteomic profiling; Table 2), but also by the identification of increased immunoreactivity of additional key players such as Siglec1 and CD68 within macrophages in sIBM muscle (see below), adding additional markers to the subsequent analyses.

**Table 2:** List of proteins altered in abundance and controlled by interferon-mediated processes (based on “Interferome” database).

**Acces-sion #**	**Peptide count/unique peptides**	**Anova (p)**	**Description**	**Gene Symbol**	**Ensembl ID**	**Fold of regu-lation [log2]**
**UPREGULATED PROTEINS**						
Q99972	1/1	0.04	Myocilin	*MYOC*	ENSG00000034971	6.49
P15153	1/1	< 0.0005	Ras-related C3 botulinum toxin substrate 2	*RAC2*	ENSG00000128340	5.21
P42224	9/9	0.01	Signal transducer and activator of transcription 1-alpha/beta	*STAT1*	ENSG00000115415	4.99
Q14764	8/8	< 0.0005	Major vault protein	*MVP*	ENSG00000013364	4.66
Q9UJU6	1/1	< 0.0005	Drebrin-like protein	*DBNL*	ENSG00000136279	4.60
P08754	1/1	0.01	Guanine nucleotide-binding protein G(k) subunit alpha	*GNAI3*	ENSG00000065135	4.44
P33241	1/1	0.02	Lymphocyte-specific protein 1	*LSP1*	ENSG00000130592	4.29
Q03519	1/1	0.01	Antigen peptide transporter 2	*TAP2*	ENSG00000204267 ENSG00000250264	3.95
O95865	2/2	< 0.0005	N(G),N(G)-dimethylarginine dimethylaminohydro-lase 2	*DDAH2*	ENSG00000213722	3.89
P32455	4/3	0.03	Interferon-induced guanylate-binding protein 1	*GBP1*	ENSG00000117228 ENSG00000225492	3.72
Q9UKY7	1/1	< 0.0005	Protein CDV3 homolog	*CDV3*	ENSG00000091527	3.65
O14879	1/1	0.03	Interferon-induced protein with tetratricopeptide repeats 3	*IFIT3*	ENSG00000119917	3.58
Q9UHD8	4/3	< 0.0005	Septin-9	*SEPT9*	ENSG00000184640 ENSG00000261843	3.58
Q14203	3/3	< 0.0005	Dynactin subunit 1	*DCTN1*	ENSG00000204843	3.46
P49756	1/1	< 0.0005	RNA-binding protein 25	*RBM25*	ENSG00000119707	3.32
Q9Y3B3	1/1	0.01	Transmembrane emp24 domain-containing protein 7	*TMED7*	ENSG00000134970	3.19
P78559	1/1	0.02	Microtubule-associated protein 1A	*MAP1A*	ENSG00000166963	3.16
Q14980	1/1	0.02	Nuclear mitotic apparatus protein 1	*NUMA1*	ENSG00000137497	3.12
P35580	9/4	0.04	Myosin-10	*MYH10*	ENSG00000133026	2.91
P23142	3/3	0.02	Fibulin-1	*FBLN1*	ENSG00000077942	2.87
Q13033	1/1	0.01	Striatin-3	*STRN3*	ENSG00000196792	2.79
O75534	1/1	0.03	Cold shock domain-containing protein E1	*CSDE1*	ENSG00000009307	2.77
Q99536	4/4	< 0.0005	Synaptic vesicle membrane protein VAT-1 homolog	*VAT1*	ENSG00000108828	2.77
Q9BSJ8	6/6	< 0.0005	Extended synaptotagmin-1	*ESYT1*	ENSG00000139641	2.76
P62070	1/1	< 0.0005	Ras-related protein R-Ras2	*RRAS2*	ENSG00000133818	2.75
Q07065	2/2	0.01	Cytoskeleton-associated protein 4	*CKAP4*	ENSG00000136026	2.75
Q9Y696	4/4	0.01	Chloride intracellular channel protein 4	*CLIC4*	ENSG00000169504	2.75
O60240	9/8	0.05	Perilipin-1	*PLIN1*	ENSG00000166819	2.73
P32456	3/2	0.01	Interferon-induced guanylate-binding protein 2	*GBP2*	ENSG00000162645	2.72
Q15075	4/3	0.02	Early endosome antigen 1	*EEA1*	ENSG00000102189	2.72
P04275	5/5	< 0.0005	von Willebrand factor	*VWF*	ENSG00000110799	2.71
P04083	9/8	< 0.0005	Annexin A1	*ANXA1*	ENSG00000135046	2.70
P51911	2/2	0.01	Calponin-1	*CNN1*	ENSG00000130176	2.66
Q9Y4L1	2/2	0.04	Hypoxia up-regulated protein 1	*HYOU1*	ENSG00000149428	2.61
P09936	5/4	0.02	Ubiquitin carboxyl-terminal hydrolase isozyme L1	*UCHL1*	ENSG00000154277	2.57
P16070	1/1	< 0.0005	CD44 antigen	*CD44*	ENSG00000026508	2.52
**DOWNREGULATED PROTEINS**						
P02585	9/9	0.01	Troponin C, skeletal muscle	*TNNC2*	ENSG00000101470	−2.25
P48788	12/12	0.01	Troponin I, fast skeletal muscle	*TNNI2*	ENSG00000130598	−2.21
P45378	21/20	0.01	Troponin T, fast skeletal muscle	*TNNT3*	ENSG00000130595	−2.19
P16219	5/5	0.02	Short-chain specific acyl-CoA dehydrogenase, mitochondrial	*ACADS*	ENSG00000122971	−1.83
P30711	3/2	0.01	Glutathione S-transferase theta-1	*GSTT1*	ENSG00000184674	−1.37
Q12797	1/1	0.01	Aspartyl/asparaginyl beta-hydroxylase	*ASPH*	ENSG00000198363	−1.11
P11217	55/42	0.01	Glycogen phosphorylase, muscle form	*PYGM*	ENSG00000068976	−1.10
Q8N335	8/7	0.01	Glycerol-3-phosphate dehydrogenase 1-like protein	*GPD1L*	ENSG00000152642	−1.10

#### Cellular Localization of CD74, CD163, and STAT1 in Skeletal Muscle Biopsies From sIBM Patients

**Figure 2:**
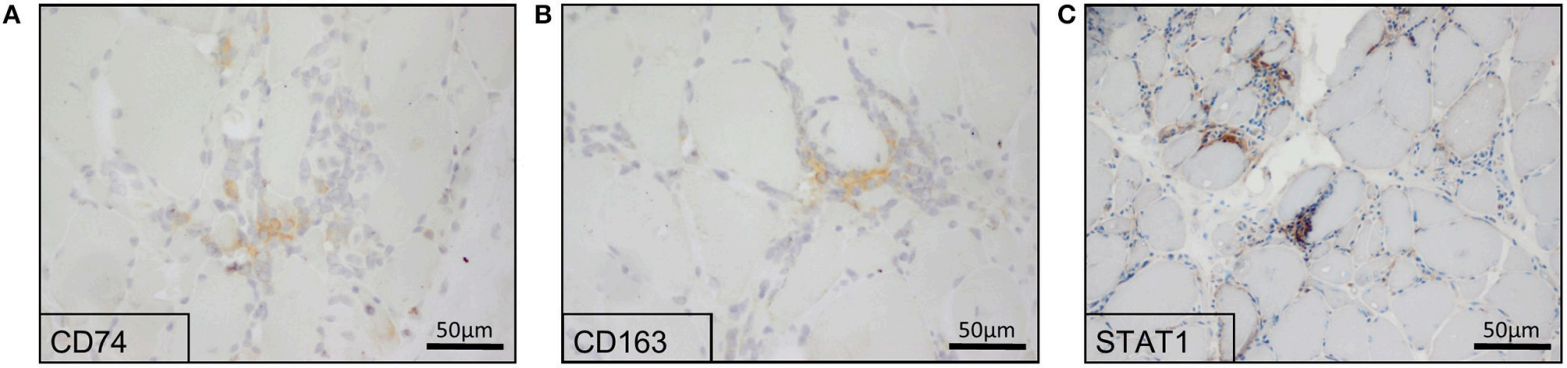
Immunohistochemical reactions of potential key players in the skeletal muscle biopsies from sIBM patients. Proteomics-based identified key players showed a positive staining in sIBM patients' skeletal muscle biopsies, where CD74 **(A)**, as well as CD163 **(B)** highlighted macrophages. STAT1 **(C)** was expressed in macrophages in active myophagocytosis, while it was not present at the sarcolemma of myofibers.

To verify the *in silico* results and the respective increased expression of CD74, CD163, and STAT1 in sIBM muscle biopsies, we stained sections of muscle biopsy specimens in a larger cohort of sIBM patients (*n* = 13). In sIBM patients' skeletal muscle biopsies, CD74 mostly stained endomysial macrophages (Figure 2A). CD163 highlighted macrophages in the endomysium but was not positive on the sarcolemma of the myofibres (Figure 2B). STAT1 was expressed in macrophages in active myophagocytosis but not in the endo- and perimysium, independently of macrophages. STAT1 antibodies did not stain the sarcolemma of myofibers (Figure 2C). In summary and on a descriptive first approach, all three molecules are detectable on a variety of macrophages in the endomysium or in myophagocytosis.

#### Significance of CD74, CD163, and STAT1 to Immunological Processes in Skeletal Muscle of sIBM Patients

**Figure 3:**
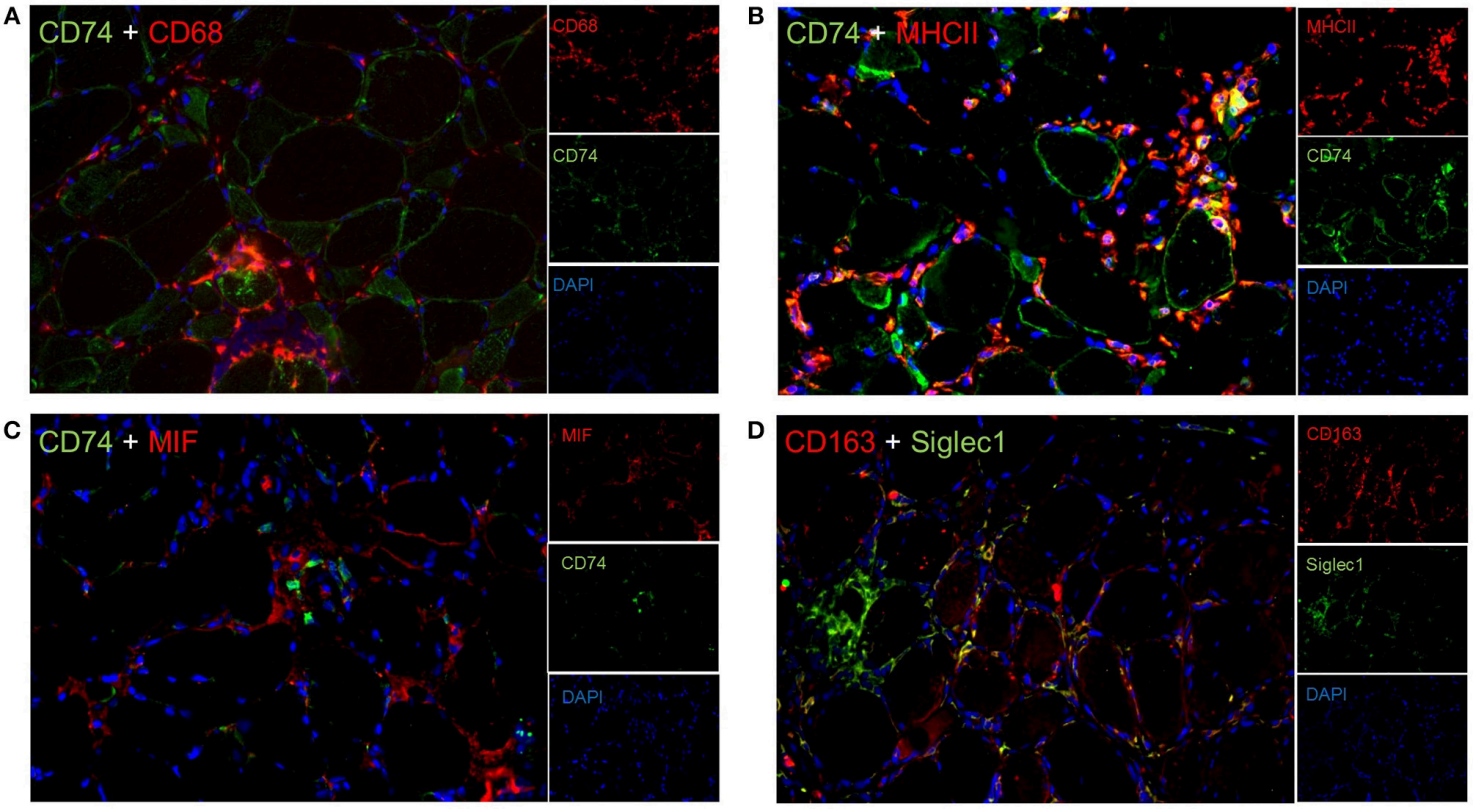
Double immunflorescent staining reveal functional interactions in sIBM patients' muscle tissue. Co-staining of various proteins revealed that CD74 co-labels with CD68+ macrophages **(A)**, and MHC class II **(B)**, but not with the macrophage migration inhibitory factor MIF **(C)**. In addition, CD163^+^ macrophages partially co-express Siglec1 **(D)**.

To expand these descriptive findings and to further implement functional association of the above-mentioned quantitatively and qualitatively highly relevant proteins, we analyzed additional proteins which are predicted to interact with those *in situ* and studied co-expression of several markers by immunofluorescence. CD74 stained some muscle fibers sarcolemmaly and co-localized with CD68^+^ macrophages (Figure 3A), as well as with sarcolemmal MHC class II immunoreactivity (Figure 3B). On the contrary, the macrophage migration inhibitory factor MIF did not co-stain with CD74 (Figure 3C). Furthermore, our immunostaining studies revealed that many CD163^+^ macrophages co-stained with Siglec1 (CD169) in the endomysium (Figure 3D).

**Figure 4:**
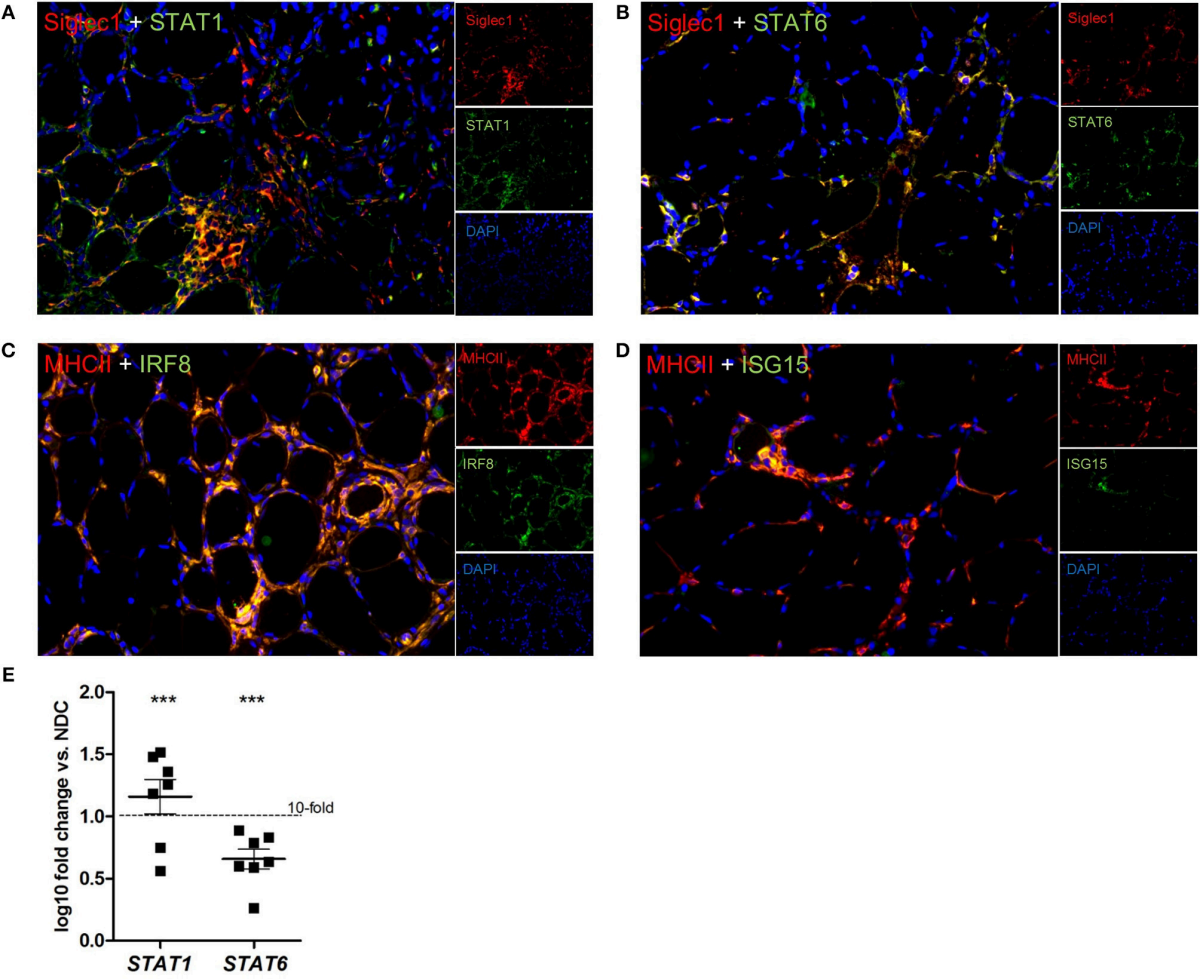
different subforms of macrophages, as well as type I interferon pathways are found in sIBM. We could demonstrate Siglec1+ macrophages, which express STAT1 **(A)**, or STAT6 **(B)**, hinting at involvement of different macrophage subtypes in sIBM muscle inflammation. The activation of STAT1 and STAT6 could also be demonstrated by significantly elevated gene expression levels **(E)**, *p* < 0.01. MHC class II+ macrophages also clearly co-stained with key proteins of the type I interferon pathway like IRF8 **(C)** and ISG15 **(D)**.

In addition, Siglec1^+^ macrophages expressed the transcription factor STAT1 in active myophagocytic clusters (Figure 4A), while STAT6 was expressed endomysially (Figure 4B), demonstrating the involvement of different yet complementary immune mechanisms in the course of sIBM-associated muscle inflammation. Importantly, both markers were also significantly elevated on the transcript level in skeletal muscle biopsies of sIBM patients as compared to NDCs (Figure 4E), with gene expression of *STAT1* being elevated around 15-fold, and of *STAT6* around 4,5-fold. Of note, activated MHC class II^+^ macrophages strongly co-stained with key proteins of the type I interferon pathway such as IRG8 and ISG15 (Figures 4C,D and Table 2).

## Discussion

In the present study, data from unbiased proteomic analysis highlighted the presence of certain proteins playing decisive roles in immune response to be highly abundant in sIBM patients' skeletal muscle biopsies. *In silico* analyses and subsequent morphological studies in skeletal muscle revealed their key characteristics in pathways driving specific macrophage responses. Along this line, Siglec1^+^ CD163^+^ and Siglec1^+^ STAT1^+^ macrophages were identified. Furthermore, MHC class II^+^ macrophages co-expressed ISG15 and IRF8 in patient-derived muscles highlighting a tight bond between these activated macrophages and the type I and type II interferon responses. Additionally, MHC class II^+^ macrophages and the sarcoplasm of adjacent muscle fibers expressed CD74.

These findings have important implications for the current understanding of the role of specifically activated macrophages in the pathogenesis of sIBM. Several ways of addressing the activation of macrophages *in vivo* and *in vitro* by using targeted and unbiased approaches notoriously exist (13, 23–27). Flexible states of macrophage activation have been found to occur according to their vast duties in physiology and pathophysiology of different diseases, specifically in chronic inflammatory and fibrotic diseases (24, 28–31). In sIBM, the vast majority of studies focusing on the immune-system have addressed the adverse effects of systemic T cell function and dysfunction or T cell dysfunction within the muscle itself (1, 5, 32–38). In contrast, only few studies have had a broader approach in tackling additional aspects of the immune response (48, 49) or genetic factors influencing the pathogenesis such as the *SQSTM1* or *VCP* variants (39). Proteomic analysis has recently helped to decipher new and unexpected molecules involved in the pathophysiology of sIBM such as FYCO1 and its role in autophagy or the composition of protein aggregates in rimmed vacuoles (40). While the latter studies have focused on the elucidation of vacuole pathophysiology, in the present study we have used the whole protein extracts of skeletal muscle biopsies to obtain molecular information about the entire tissue, and thus, to gain a better and unbiased understanding of the etiology of the disease. This approach revealed that CD74, CD163 and STAT1, driving inflammatory responses, were at the forefront of the highly expressed proteins within the diseased skeletal muscle tissue. This finding accords with our hypothesis of a major role for molecules driving macrophage polarization since sIBM biopsies feature very strong immunity-related aspects over all with macrophages being by far the most abundant cell type in the lesion. Hence, we were able to characterize the immunity of these macrophages in more detail and found molecules identified by proteomic analysis and subsequently verified by immunohistochemistry in biopsies which are strongly related to type I/II interferon responses, specifically IRF8, ISG15, GBP1, GBP1P1, HLA-DOB, IFIT3, STAT1, TAP2. Since macrophages can adopt a great variety of functional states (24, 31, 41, 42), it was not surprising to identify a further important subgroup consisting of STAT1^+^Siglec1^+^ macrophages in active myophagocytosis. This type of macrophages is implicated in acute clearance of necrotic muscle fibers, a process which, despite the explicitly chronic character of the disease (active over decades), is a constant muscle biopsy feature. Of note, we also identified STAT6^+^Siglec1^+^ macrophages in the endomysium at distance of myophagocytic and necrotic fibers, highlighting that both downstream transcription factors may become activated in certain macrophages, which have then different duties and fates. A specific immune phenotype of Caucasian sIBM patients with HLA-A3 suggesting MHC class I activation has been described (43), MHC class II staining patterns were recently described for sIBM patients in comparison to dermatomyositis and anti-synthetase syndrome-associated myositis (44). This finding is in keeping with our proteomic identification of elevated CD74, as a protein regularly interacting with MHC class II molecules. Our findings also indicate that macrophages (which are mentioned as being the most abundant mononuclear inflammatory cells in skeletal muscle biopsies of sIBM patients) have been underestimated as cellular key players in the etiopathology of sIBM. The second relevant finding of this study, is the type I and type II interferon response identified both by proteomic profiling, subsequent *in silico* studies, and immunohistochemistry, expanding previous data where the interferon signature genes have been addressed by transcriptomics (45). Notably, the signatures of type I and type II Interferons, identified here, differ from the ones that have been described to be crucial in dermatomyositis (45–47). Herewith, the relevant role of macrophages as versatile multifunctional immune cells playing a decisive role in the etiopathology of sIBM was shown. These findings may define a molecular starting point for future therapeutic approaches in sIBM utilizing JAK-STAT inhibitors.

## Summary

In summary, we demonstrate that unbiased proteomic profiling of skeletal muscle biopsies provides important insights into the molecular etiology of a disease and more precisely in the context of sIBM revealed proteins prominently involved in immunity and characterizing a pattern of macrophage activation. Application of immunohistochemical verification and analysis of these cells in the context of the type I and type II interferon signature *in situ* allowed the attribution of these proteins to specific functional states of macrophage activation. Hence, we show that several different types of macrophages are actively affecting the immune response in sIBM, via a prominent type I interferon signature among others.

## Ethics Statement

This study was carried out in accordance with the recommendations of Charité ethics committee with written informed consent from all subjects. All subjects gave written informed consent in accordance with the Declaration of Helsinki. The protocol was approved by the Charité ethics committee. No. EA2/163/17.

## Author Contributions

AR and CP: design of the study, acquisition, analysis and interpretation of data, as well as drafting the manuscript. DH: acquisition and analysis of data, as well as revising the manuscript. H-HG: design of the study and revising the manuscript. WS: design of the study, interpretation of data, as well as drafting the manuscript.

### Conflict of Interest Statement

The authors declare that the research was conducted in the absence of any commercial or financial relationships that could be construed as a potential conflict of interest.
